# The Value of Cognitive Pretesting: Improving Validity and Revealing Blind Spots through the Development of a Newborn Screening Parent Experiences Survey

**DOI:** 10.3390/ijns7030041

**Published:** 2021-07-08

**Authors:** Norma-Jean Simon, Anne Atkins, Brianne Miller, Natasha Bonhomme, Beth Tarini

**Affiliations:** 1 Center for Translational Research, Children’s National Research Institute, Children’s National Hospital, Washington, DC 20010, USA; nsimon@childrensnational.org (N.-J.S.); aatkins@childrensnational.org (A.A.); bcmiller@childrensnational.org (B.M.); 2 Expecting Health, Washington, DC 20008, USA; nbonhomme@geneticalliance.org; 3 Department of Pediatrics, School of Medicine and Health Sciences, The George Washinton University, Washington, DC 20052, USA

**Keywords:** newborn screening, survey development, cognitive interviews

## Abstract

Surveys are used to gather a range of data on newborn screening (NBS) processes. We describe the development of a survey about parents’ NBS experiences, in the United States, informed by cognitive pretest interviews among parents with varying NBS test results (true-positive, false-positive, normal). Cognitive pretest interviews were conducted following a semi-structured script and notes were taken to identify problematic survey items. The study team met weekly to discuss pretest feedback, draft changes, and generate revised items. Pretests indicated that parent experiences with NBS are varied and NBS screening procedures are not well understood. Substantial modifications were made to survey questions concerning NBS testing and result communication. Pretesters often associated NBS with other tests/exams/scales—APGAR scores, Ages and Stages questionnaires, and genetic testing during pregnancy. Some pretesters recalled receiving NBS blood spot results during their hospital admission, an uncommon practice, and few recalled knowing results would be provided to them or their pediatrician in the first few weeks of life. Thorough explanations regarding NBS procedures and expectations were embedded within the survey to enhance and improve interpretation of survey questions. Future NBS experience surveys should utilize cognitive pretesting to capture divergent experiences and improve response validity.

## 1. Introduction

Surveys are commonly used to gather a range of data on newborn screening (NBS) programs. In recent years, surveys have been employed to evaluate and improve NBS program processes including laboratory procedures [1], hospital blood spot collection [2], NBS results and follow-up communication [3,4,5,6], and parent education strategies [7,8,9]. An evolving area of research in newborn screening is the evaluation of public perceptions and parental knowledge and experiences with NBS [10,11,12,13]. In this vein, we created a survey to examine NBS experiences among parents including recall of NBS testing, receipt of NBS results, and NBS communication experiences with providers.

While surveys are a valuable tool to gather parent experiences, ensuring data gathered from surveys are reliable and valid presents a challenge. Cognitive interviewing is an evidence-based method of assessing if a survey gathers the data investigators intend [14,15,16,17]. Cognitive interviews allow investigators to: test for construct validity of new items; improve survey item relevance to participants; identify undisclosed assumptions that may unintentionally affect survey response; and aid in the analysis and interpretation of results [18].

We describe here the development of a survey to solicit parental experiences and perceptions regarding NBS processes and procedures after the birth of a child. We demonstrate the utility of cognitive pretest interviews as a means of evaluating survey validity and describe survey revisions made, incorporating pretest feedback, to improve the quality of survey responses.

## 2. Materials and Methods

We assembled a team of NBS experts, survey method experts, and research staff to develop a NBS parent experiences survey. The survey was developed as part of a National Institutes of Health (NIH) funded NBS outcomes study in the United States (see funding statement). The aim of the eventual case–control study is to understand parent experiences receiving false-positive NBS results and assess long-term effects on parental stress, anxiety, and health seeking behaviors. We included the following domains in the survey: family demographics; pregnancy and birthing experiences; child health; parental mental health; and NBS experiences including recall of testing, NBS results, and provider communication. An initial draft of the NBS parent experience survey was developed over a 6-month period. Where possible, we incorporated or adapted validated survey instruments [19,20,21,22,23,24,25,26]. When an existing instrument was not available, we drafted new survey items. Iterative drafts of the survey were circulated to NBS and survey experts to refine domain and question content. Once a complete initial survey was developed, a research analyst built the survey in a REDCap database [27,28], allowing the study team to continue survey development and conduct rapid, iterative testing cycles.

### 2.1. Cognitive Pretest Recruitment

A convenience sample of parent pretesters in the United States were invited to participate in cognitive interviews between November 2019 and May 2020. Initial pretesting candidates were recruited among existing contacts of the study team. Subsequent candidates were recruited through NBS program coordinator collaborators to test the survey with a population that more closely resembled future participants. Pretesters were recruited to participate if they had a child <24 months of age. Efforts were made to recruit parents of children ≤6 months of age to examine NBS questions that required recent recall. Upon recruitment, interested pretesters were directed to reach out to a member of the study team to schedule a cognitive pretest interview. The study team informed parents of the study purpose and the format of cognitive pretest interviews via an introductory phone call or e-mail. Prior to the scheduled cognitive interview, parents were sent personalized survey links to review. All cognitive interviews were conducted by phone and lasted 30–60 min. All individuals participated in only one cognitive pretest interview. Recruitment and pretesting procedures were reviewed and determined not regulated by Children’s National Institutional Review board.

### 2.2. Cognitive Pretest Interviews

Cognitive pretest interviews included testing the newly developed NBS parent experiences survey including questions on the domains described above as well as validated questions on demographics [19], child health [20], provider communication [21], and health literacy [22]. In addition, we tested a battery of validated instruments including: age-specific Ages and Stages questionnaires [23], the Parental Stress Index [24], Vulnerable Baby Scale [25], and the PROMIS^®^ anxiety scale [26]. The validated survey battery was tested on a subset of pretesters to assess acceptability for a planned outcomes study and inform the interpretation of future analyses.

All pretests followed a semi-structured script with prompting questions to elicit feedback on readability, interpretability and comprehension of survey items. Initially, we asked participants to take the survey in a live format by phone in which probing questions were asked as the participant took the survey (Table 1). Notes were taken during cognitive interviews concerning survey items flagged for review. The study team met weekly to discuss pretest feedback, draft changes to the survey, and generate items and areas of focus for subsequent pretests with new participants. Clinical and survey experts were consulted as needed. After several pretests were conducted in a live format, we modified our pretest strategy to ask participants to review and complete the survey prior to the scheduled cognitive interview. This allowed the study team to ask questions regarding the experience of taking the survey in conditions similar to those of future study participants, online and independently, and allowed the team to allocate more time on targeted questions related to areas of focus. Cognitive interview pretest survey data were deleted and not retained for future analysis. Pretesters were compensated for participation.

Survey pretesting and revisions focused on (1) readability and interpretation of plain language definitions; (2) comprehension of NBS testing items; (3) recall and comparability of NBS result communication; and (4) general feedback including survey applicability, accessibility, appearance, and flow. We provide a summary of pretest feedback and illustrative examples of revisions informed by cognitive interviews.

## 3. Results

We conducted 28 cognitive pretest interviews among 22 unique families with varied recall of NBS testing and results (11 mothers; 4 fathers; 6 mother-father pairs; and 1 other caregiver). Of participating families, 11 recalled receiving within-range or normal results, 1 parent recalled false-positive results, and 9 families recalled receiving true-positive results. One participant was a non-primary caregiver recruited specifically to test bilingual demographic questions and was not asked about NBS results. Domains of the parent experience survey were developed and refined throughout the cognitive pretesting period. Early pretests focused on refining plain language definitions of medical terms and survey flow. Subsequent pretests focused primarily on construct validity and comparability of the NBS testing, result, and provider communication items. While all domains of the NBS experiences survey underwent revision, substantial modifications were made to the NBS testing, results and communication items.

### 3.1. Plain Language Definitions

Given the general medical history information collected within the NBS experiences survey, we aimed to test a number of plain language definitions to describe common pregnancy and birthing procedures and complications. Definitions were tested for uniformity of understanding across participants with different parenting roles. Pretesters were not always familiar with medical terminology in general or with a specific medical procedure or complication; however, pretesters were forthcoming with feedback on medical terminology—both supportive and critical. When asked about terminology for common complications during pregnancy such as high blood pressure, gestational diabetes, and urinary tract infections, most pretesters indicated being familiar with the term or described that the plain language definition was sufficient to understanding if the procedure or complication was experienced. More often, pretesters indicated when specific terminology created confusion. For example, terms such as injury or vaginal bleeding were confusing to pretesters. In both these cases, presters noted that these words were non-specific and poorly defined. This feedback allowed us to better refine terminology and plain language definitions so participants felt confident in evaluating their experience with the discrete response categories provided.

### 3.2. Newborn Screening: Testing Experiences

Pretest participants described, through their own experiences, that NBS processes, procedures, and expectations are not necessarily well understood. Many pretesters initially conflated NBS with other tests such as APGAR scores, Ages and Stages questionnaires, and genetic testing during pregnancy. Once clarification was provided, parents reported a variety of experiences with learning about and witnessing NBS tests shortly after birth. Given parents’ varying experiences, prior communications, and knowledge of NBS, we tested terms such as PKU test, heel-prick test, and blood spot to evaluate which terms resonated most with pretesters.

**Figure 1:**
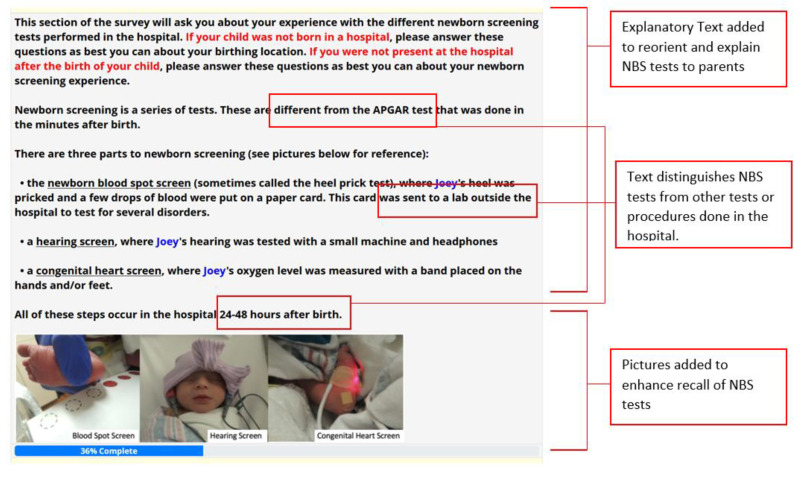
Newborn Screening Introduction Text Revisions based on Pretest Feedback.

Our goal was to identify ways to better orient participants to the NBS questions and improve NBS recall. As such, we created a focused introduction section before the NBS survey specific questions to provide an overview of NBS processes, procedures, and expectations to ensure similar baseline knowledge and orientation to NBS (Figure 1). We included explanatory text with explicit mentions of the tests referenced by pretest participants that are not considered part of newborn screening. We also included pictures of NBS testing procedures to enhance recall of the NBS tests with considerations for the timing of test collection. Parents indicated that recall was aided with pictures.

**Figure 2:**
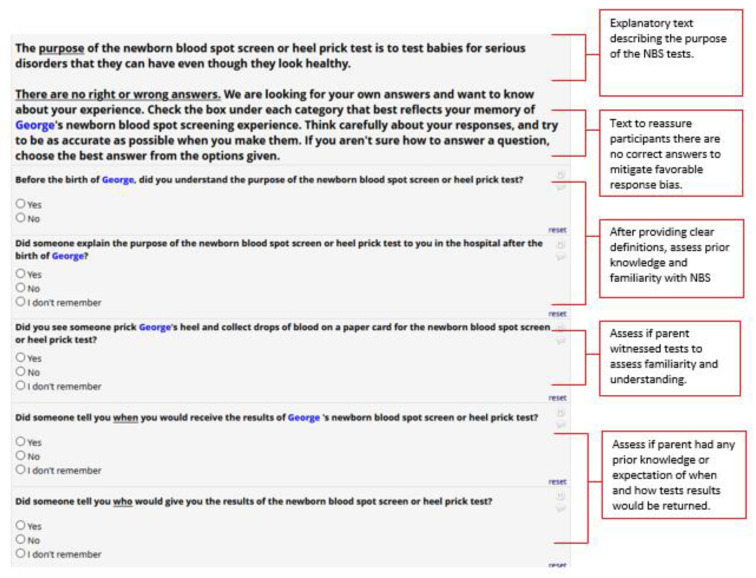
Newborn Screening Test Example Questions after Revision.

To further aid recall and response of NBS events, we developed several specific questions based on expectations of what parents would most often experience in the hospital related to newborn screening (Figure 2). These questions were replicated for each of the NBS tests (hearing, congenital heart screen, and blood spot test) and included (1) if the parent understood the purpose of the NBS test, (2) if the purpose of the test was explained in the hospital, (3) if the parent saw the test being conducted or the parent was told that the test had been conducted, (4) the result of the test, and (5) communication on when and by whom the results of the test would be delivered. Upon pretesting with respondents, we found that asking these series of questions improved recall of past NBS experiences, and better prepared participants to assess their recollection of NBS result communication.

### 3.3. Newborn Screening: Result Communication Expectations

**Figure 3:**
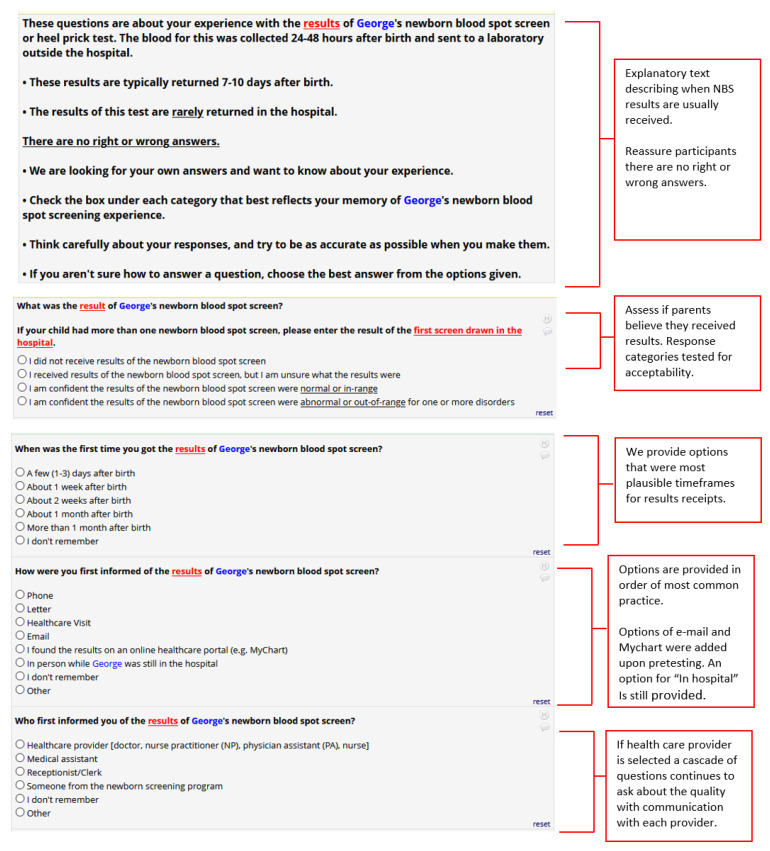
Newborn Screening Result Communication Example Questions.

During pretest interviews, many parents recalled either having received or having had an expectation of receiving NBS blood spot results within the hospital, a relatively uncommon practice. We noted that some pretesters were inclined to answer positively that they remembered an NBS test or result in their survey response, because “no news is good news”, even when they did not necessarily remember or recall the NBS test or specific communication about the result. Thus, to accommodate for the perceptions of where and when parents would likely receive NBS results, we included text to explain typical program expectations regarding where NBS blood spots are processed and how results would most likely be communicated. We also added an explicit direction that results are rarely returned in the hospital. Finally, we adjusted our response categories from dichotomous “Yes/No” response categories to categories that echoed parents’ responses during cognitive interviews of how parents evaluated receiving or recalling the results of NBS tests (Figure 3). After these changes, we noted that parents appeared more comfortable indicating that they either did not receive results or were not sure if they received results in subsequent pretests.

### 3.4. Newborn Screening: Provider Communication

A subset of cognitive pretest interviews was conducted among parents who reported receiving false-positive and true-positive NBS results to test questions regarding follow-up provider communication experiences. We designed this section of the survey to capture all communication experiences that a parent may have receiving results: from an initial phone call to an in-depth experience speaking with a genetic counselor or specialist health care provider. We tested several question cascades to capture NBS communication experiences and asked parents to rate each conversation they had with a provider regarding NBS results. 

We found that parents who recalled receiving false-positive or true-positive results would indicate having conversations with three or four providers over multiple interactions. Parents indicated that when rating satisfaction with provider communication, they did not think about each individual interaction or conversation, but rather they rated their satisfaction with each provider’s communication style overall. We therefore asked parents to rate their overall communication with each provider rather than each conversation and further inquired how many conversations a parent had regarding NBS screening. Parents often proceeded to assess providers they had NBS communication with sequentially until they accessed a provider that communicated their NBS screening results to their satisfaction.

### 3.5. Overall Survey Applicability, Accessibility, Appearance, and Flow

Finally, we tested the overall acceptability, time burden, and survey navigation with pretesters. Pretesters reported completing surveys on multiple electronic devices including tablets, phones, and computers. The NBS experience survey took 20–30 min to complete. Mother-Father pairs reported taking surveys independently, but that they also occasionally consulted with one another. We therefore added text affirming that surveys are intended to be taken independently in the introductory text of the survey.

Pretesters suggested that a progress bar, particularly on the validated battery, would better guide completion. A progress bar was subsequently added along with anticipatory text to guide the participant. Other visual elements added and tested included a study logo, color formatting, and generous use of underlined and piped text to make questions more relevant to participants.

We were mindful of the potential for participant burden given the length of our NBS parent experiences survey. We wanted to balance burden with the opportunity for all parents to provide their experiences related to the pregnancy and delivery of their newborn, important context for understanding a parent’s NBS experience. We pretested survey items related to pregnancy and delivery with non-birthing parents; if non-birthing parents were neither knowledgeable nor comfortable with these survey items, we intended to restrict these domains to the birthing parent only. We found that non-birthing parents, all of which were fathers in our sample, responded with a high level of confidence when answering questions relating to pregnancy and delivery. Given this feedback we proceeded to survey non-birthing parents about pregnancy and delivery, with an addition of a “don’t know” option.

Finally, in a subset of parents, we tested a validated battery of surveys intended for administration with parents every 6 months for a future outcomes study. These validated batteries took 30–45 min to complete, a longer time period than expected. While we did not aim to modify any validated survey items, we noted the expected time burned for completion and readjusted our incentive structure to accommodate this. In addition, we flagged questions on validated batteries that were challenging for parents to assess. In particular, upon the onset of the global COVID-19 pandemic, parents indicated struggling with questions related to parental stress, anxiety, and child health. One pretester noted that her responses regarding allowing individuals to interact with her child in and outside of her home as well as questions regarding her own feelings of isolation, parenting support, and stress are very different pre- and post-pandemic. Parents thus struggled with answering questions with regard to their “normal” parenting behavior in contrast to their “COVID” parenting behavior. To account for the potential impact of the pandemic on parenting behavior, we added questions on the repeated battery related to parent worry about the COVID-19 pandemic that could be tracked over time and aid in our interpretation of results. 

## 4. Discussion

Cognitive pretest interviews revealed important insights into the varied experiences parents have with NBS. Importantly, pretesters’ feedback resulted in substantial modifications to an NBS parent experiences survey. Nearly all domains of the NBS parent experiences survey underwent revision through rapid, iterative cycles of cognitive pretesting. Notable modifications were made to revise plain language definitions, clarify instructional text regarding NBS processes, procedures and expectations, and facilitate survey navigation with the addition of visual graphics. Cognitive interviews improved the reliability and validity of survey items and will be used to further inform the interpretation of results of a forthcoming NBS outcomes study.

While we set out to conduct pretest interviews to ensure we captured variability in parent NBS experiences and result communication, experiences diverged more than the survey development team had expected. As a result, we conducted more cognitive pretests than originally planned, delaying survey development completion. Planning for and allocating sufficient time for pretesting activities is recommended and important for fielding a survey that a study or program team is confident will gather reliable and interpretable data. Most importantly, pretest interviews revealed divergent baseline knowledge and recall of NBS events among parents. This finding is aligned with previous literature calling for improved NBS education to address new and persistent gaps in parental knowledge and education [7,8,12]. While NBS is a universal program in which nearly all babies are screened upon birth in the United States, NBS testing is not conditional on parent consent and understanding [29]. Research from other countries also indicate a lack of parental understanding of NBS even when informed dissent or consent processes are employed [30]. It is therefore plausible that that families may frequently participate in NBS programs without a complete understanding of NBS program purpose, processes, or procedures. While guidelines exist for NBS parent and provider education, no national standard exists in the United States. As a result, states and territories ultimately manage their own NBS program and set their own NBS priorities in collaboration with a diffuse network of birthing facilities and primary care providers that are the most proximal NBS communicators to parents and families.

The complex nature of the state-based NBS system in the United States and the various points in which parents and families may learn about NBS poses a significant challenge to constructing a survey on NBS parent perceptions and experiences. Cognitive pretesting the NBS parent experiences survey helped us appreciate the varied administration of NBS programs across US states and the impact of this on families. It is challenging to survey families about a topic in which no single entity is responsible for program education, coupled with the common experience in which families may interact with NBS only briefly with no concerning result. Thus, in revising our NBS experiences survey, we modified items with the assumption that many parents may have minimal familiarity or recall of NBS events. We aimed to identify common processes and procedures conducted across states to set expectations regarding common NBS experiences. We found providing baseline educational and explanatory text ensured that survey participants would have similar knowledge against which they could anchor and evaluate their own NBS experiences. The need to provide baseline knowledge to survey participants is consistent with a recent study by Evans et al. which found that while 62% of parents indicated some familiarity with NBS, only about one-third of parents could accurately select a definition of NBS [9].

Furthermore, considering our survey will be deployed to examine differing experiences and longitudinal outcomes contingent on NBS screening result, we aimed to understand a parent’s certainty that a NBS test happened to fully elicit the experience of receiving NBS results. One significant issue that challenged our ability to assess a parent’s understanding of receiving NBS results is the common mantra: “No news, is good news.” We found that pretesters would sometimes conflate the absence of receiving direct communication about NBS testing or results as receipt of normal or within-range results. There is an element of social desirability response bias here which obscures the true nature of NBS testing and result communication experiences important for our study. While web-based surveys are viewed as mitigating social desirability bias to some degree [31], we still found this to be a challenge. To overcome this challenge, we tailored response categories for NBS survey items drawn directly from responses and explanations provided by pretest participants to replace dichotomous (yes/no) categories to better reflect natural response that parents provided upon further probing about NBS testing and results communication.

An additional insight from the cognitive pretest interviews echoed by many of our pretest participants was that parents neither felt prepared nor knew how to interpret follow-up phone calls notifying them of the need for repeat testing when tests results were in an abnormal result range, consistent with findings from previous studies [3,32]. In order to better understand and capture the depth of newborn screening experiences, particularly among parents receiving false-positive results, the study team aims to further examine NBS experiences specific to these parents. Key informant interviews will focus on parents receiving repeat NBS testing, with the intent to make parent-informed recommendations that have implications for communicating false-positive and true-positive results.

Finally, our cognitive pretest interviews were limited by the individuals we were able to recruit. Foremost, we recruited pretest candidates through contacts of the study team, which introduces selection bias and may limit the representativeness of the feedback received by pretesters. We aimed to balance this by advertising and disseminating our pretesting opportunity though NBS program coordinator collaborators. Furthermore, while we aimed to recruit pretesters with varying NBS result experiences, only one pretester recalled the experience of receiving false-positive results. In developing our survey, we drew from the experiences of parents recalling true-positive results to tailor questions on result communication for parents and families receiving false-positive results, which may not be fully generalizable. Engaging parents or public representatives relevant to the study populations through research advisory boards or patient and public involvement (PPI) methods can be a strategy to better ensure representativeness through the survey development process [33]. Finally, our survey could have benefitted from a more robust comparability assessment of survey items between differing socio-demographic groups and English and non-English speakers. Spanish is the second most widely spoken language in the United States; as such, we developed a Spanish version of the survey translating all instruments by a certified translator and pretesting sections of the survey with a native-Spanish speaker for acceptability. We recommend that pretesting efforts be intentional in terms of the comparability assessment of all language and socio-demographic groups, where possible.

## 5. Conclusions

Cognitive pretest interviews revealed varied and unexpected newborn screening experiences that challenged us to tailor a survey for a range of NBS experiences. In particular, we aimed to create our survey to accommodate and anticipate that parents may have minimal or incomplete knowledge of NBS processes. Future NBS surveys should utilize cognitive pretesting to improve response validity.

## Figures and Tables

**Table 1:** Examples of general and specific probing questions to assess readability, interpretability, and comprehension of survey items.

General Probes	
**Probes for each survey section:**	
	*Talk me through what you are thinking about as you take the survey.*
	*Did you find any questions difficult to answer?*
	*Which of the questions were you not sure how to answer?*
	*What was your overall impression of this section of the survey?*
**Probes for pauses or hesitation during a survey response:**	
	*What were you thinking about as you were answering this question?*
	*Do any of these categories seem to restrict your answer? Are there too many options to choose from?*
	*Are there any choices that aren’t here that would better match your answer?*
**Specific Probes**	
**Survey Introduction and Pretesting Experience**	
	*What questions do you have about the study? About pretesting?*
	*When you first open the survey, you see a logo associated with our study. When you see the logo, what comes to mind?*
	*How well do you think the logo represents the study?*
	*How could we better describe the study to the survey taker?*
**Survey Eligibility and Enrollment**	
	*When I asked you about [TPN, blood transfusions], what did you think about? What came to mind?*
	*What feedback do you have on the order that we ask these questions?*
	*Do you think you understood the study the way we described it? How would you describe the study to someone?*
	*Do you have any suggestions that might help us improve how we ask participants how they would like to receive the survey?*
**Pregnancy and Delivery**	
	*Were you already familiar with the medical conditions listed here?*
	*Were there any conditions that you weren’t sure how to answer because you didn’t know exactly what the term meant?*
**Child-Wellbeing**	
	*We ask a number of questions about how healthy your baby is at the end of the first section. Can you talk me through what you were thinking about as you answered these questions?*
	*We include a list of conditions or health problems that you could have been told your child has:* *Are there any conditions on this list that you aren’t sure how to answer?* *How would you describe or categorize your child’s condition?*
**Newborn Screening**	
	*When we first introduced newborn screening in this last section, what were you thinking about?*
	*Where did you first learn about newborn screening?* *What do you remember about the [newborn bloodspot, congenital heart screen, hearing] test in the hospital?* *Tell me about when you first learned about newborn screening tests? How were these tests explained? Is there a difference to being “told” that you had a test vs being “explained” that you had a test?* *Tell me about how learned about the results of the newborn screening tests.*

## Data Availability

The data presented are available on request from the corresponding author. The data are not publicly available due to participant privacy and confidentiality.
